# Aberrant dentato‐rubro‐thalamic pathway in action tremor but not rest tremor: A multi‐modality magnetic resonance imaging study

**DOI:** 10.1111/cns.14339

**Published:** 2023-07-05

**Authors:** Xiaojie Duanmu, Jiaqi Wen, Sijia Tan, Tao Guo, Cheng Zhou, Haoting Wu, Jingjing Wu, Zhengye Cao, Xiaocao Liu, Jingwen Chen, Chenqing Wu, Jianmei Qin, Luyan Gu, Yaping Yan, Baorong Zhang, Minming Zhang, Xiaojun Guan, Xiaojun Xu

**Affiliations:** ^1^ Department of Radiology, The Second Affiliated Hospital Zhejiang University School of Medicine Hangzhou China; ^2^ Department of Neurology, The Second Affiliated Hospital Zhejiang University School of Medicine Hangzhou China

**Keywords:** action tremor, essential tremor, Parkinson's disease, probabilistic tractography, quantitative susceptibility mapping

## Abstract

**Aims:**

The purpose of this study was to clarify the dentato‐rubro‐thalamic (DRT) pathway in action tremor in comparison to normal controls (NC) and disease controls (i.e., rest tremor) by using multi‐modality magnetic resonance imaging (MRI).

**Methods:**

This study included 40 essential tremor (ET) patients, 57 Parkinson's disease (PD) patients (29 with rest tremor, 28 without rest tremor), and 41 NC. We used multi‐modality MRI to comprehensively assess major nuclei and fiber tracts of the DRT pathway, which included decussating DRT tract (d‐DRTT) and non‐decussating DRT tract (nd‐DRTT), and compared the differences in DRT pathway components between action and rest tremor.

**Results:**

Bilateral dentate nucleus (DN) in the ET group had excessive iron deposition compared with the NC group. Compared with the NC group, significantly decreased mean diffusivity and radial diffusivity were observed in the left nd‐DRTT in the ET group, which were negatively correlated with tremor severity. No significant difference in each component of the DRT pathway was observed between the PD subgroup or the PD and NC.

**Conclusion:**

Aberrant changes in the DRT pathway may be specific to action tremor and were indicating that action tremor may be related to pathological overactivation of the DRT pathway.

## Graphical abstract

The DRT pathway is documented as a potentially promising underpinning for action tremor, but the comprehensive study of DRT pathway in action tremor is insufficient. This study discovers that aberrant changes in the DRT pathway may be specific to action tremor and were indicating that action tremor may be related to pathological overactivation of the DRT pathway.

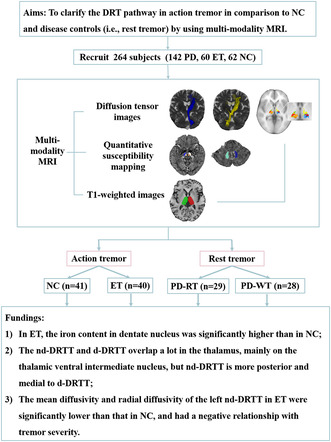

## 1 INTRODUCTION

Action tremor is one of the most frequent movement disorders observed in clinics, which is seen in essential tremor (ET).^1^, ^2^ The perturbation of the dentato‐rubro‐thalamic (DRT) pathway is documented as a potentially promising underpinning for action tremor by animal experiments and autopsy, that is to say, with an unknown negative impact on the afferent to the dentate nucleus (DN), a reduced inhibitory effect of γ‐aminobutyric acid (GABA) on DN would overactivate the whole DRT pathway passing through the red nucleus (RN) and thalamus (TH), finally leading to limb tremor,^3^, ^4^, ^5^ which is the common and central content of the mainstream hypothesis of ET action tremor, namely the GABA hypothesis and the degenerative hypothesis. However, this hypothesis is mainly based on animal experiments and autopsy, but more in vivo evidence is needed to further verify the clinical application value of the DRT pathway.

Magnetic resonance imaging (MRI) can quantify the intrinsic composition of neural pathways in vivo, which has also been widely used in the study of the DRT pathway in action tremor in recent years. The most studied component of the DRT pathway is the DRT tract (DRTT), which can be robustly tracked by diffusion tensor imaging (DTI), suggesting abnormal changes in DRTT in patients with action tremor.^2^, ^6^, ^7^ Recently, it has been found that DRTT is not a simple fiber bundle from DN to TH as previously understood, but includes the decussating DRTT (d‐DRTT) and non‐decussating DRTT (nd‐DRTT).^8^ However, d‐DRTT and nd‐DRTT have not been analyzed simultaneously in any study of action tremor. In addition, DN, as a driving hub among DRT pathway, is rarely elucidated at present, possibly because traditional MRI loses signal of DN in the cerebellum. In recent years, quantitative sensitivity mapping (QSM) has been applied to analyze the iron content of DN in Parkinson's disease (PD) patients with tremor, and it has been found that excessive iron deposition in DN is related to tremor.^9^ However, the tremor symptoms of the patients in previous studies included both action and rest tremors, so it is unclear whether excessive iron deposition in DN is specific to action tremor. Taken together, the comprehensive study of the DRT pathway in action tremor is insufficient, and whether the DRT pathway is the specific neural pathway basis of action tremor needs to be further studied.

To solve the above problems, we comprehensively quantified the components of the DRT pathway by multi‐modality MRI to investigate the role of the DRT pathway in action tremor, including quantifying the iron deposition and volume changes in nuclear in the pathway using QSM and T1‐weighted images and obtaining the diffusion parameters of DRTT using DTI. In addition, to confirm whether changes in the DRT pathway are specific to action tremor, we also included patients with rest tremor—reflecting the effect of rest tremor by comparing between subgroups of PD patients—to see whether the changes in the DRT pathway are consistent between the two types of tremor.

## 2 MATERIALS AND METHODS

### 2.1 Subjects

This research was approved by the Medical Ethics Committee of the Second Affiliated Hospital of Zhejiang University School of Medicine. All the ET and PD patients were recruited from the neurological clinics in the Second Affiliated Hospital of Zhejiang University School of Medicine, while the normal controls (NC) were recruited from the social community in Hangzhou, China. All of them participated in this study from August 2014 to November 2020, and signed informed consent form. Each patient was diagnosed by an experienced neurologist based on the following criteria: ET diagnosis based on the Movement Disorders Society Statement,^10^ and PD diagnosis based on UK Parkinson's Disease Society Brain Bank criteria.^11^ Basic demographic information, such as age, sex, education, disease duration, and neurologic and psychiatric scales including Unified Parkinson's Disease Rating Scale (UPDRS), and Mini‐Mental State Examination (MMSE) scores were obtained from all patients. Among these, the UPDRS is widely used in clinical research and efficacy evaluation of PD and includes four main domains: mental, behavioral and emotional, daily living, physical examination and treatment complications.^12^ The higher the score, the more severe the degree of dysfunction. And the MMSE is the most commonly used scale to assess general cognitive function, and the more severe the cognitive decline, the lower the score.^13^ In addition, the Essential Tremor Rating Scale (TETRAS) scores were gained from ET patients, and the Hoehn–Yahr stages were acquired from PD patients. Among them, the TETRAS was developed by the Tremor Research Group (www.tremorresearchgroup.org) to evaluate the severity of ET, with higher scores representing more severe symptoms. And the Hoehn–Yahr stages are currently a relatively well‐recognized PD staging system, with a total of 0–5 stages, with severity increasing with level.^14^ For PD patients treated with medication, clinical assessments, and image scanning were performed on individuals receiving medication after they had stopped using all antiparkinsonian medications for the night (at least 12 h).

Sixty ET patients, 142 PD patients, and 50 NC patients were initially recruited in the study. According to the following exclusion criteria, 9 ET, 37 PD, and 9 NC were excluded: (1) left‐handed or double‐handed; (2) history of neurologic or psychiatric disorders (e.g., severe brain atrophy, brain injury, brain surgery, cerebrovascular disease, schizophrenia, emotional disorders); (3) significant cognitive decline (MMSE scores ≤17 for illiterate subjects, ≤20 for grade‐school literate subjects, and  23 for junior high school and higher education literate subjects);^15^, ^16^ and (4) severe metal dentures and motion artifact (Figure S1).

In order to specifically study the role of action tremor, we further screened ET patients without rest tremor of both upper limbs (UPDRS‐20 any upper limbs scores ≥1 were excluded, *n*_ET_ = 11; Figure S1). Because our action tremor scale only assessed symptoms in the upper limbs,^12^ we screened PD patients with rest tremor only in the upper limbs for better comparison. The PD group was divided into PD with rest tremor (PD‐RT) (UPDRS‐20 has at least one upper limbs scores ≥2, *n* = 43) and PD without rest tremor (PD‐WT) (UPDRS‐20 double upper limbs scores = 0, *n* = 46).^17^ Because PD patients often have both action tremor and rest tremor,^18^, ^19^ there were only 7 PD‐WT and 6 PD‐RT have no action tremor. Considering the effect size of the study, we adopted an alternative method to specifically highlight the effect of the rest tremor in PD patients as follows. We selected two PD subgroups by using a propensity score matching (PSM) method to match patients 1:1 in terms of sex, age, and other motor scores [action tremor, akinetic/rigid (AR), and postural instability/gait disorder (PIGD)];^20^ subsequently, 29 subjects were obtained from each group (PD‐RT and PD‐WT), and finally, one PD‐WT patient was excluded for the failure of QSM preprocessing (Figure S1).

Finally, 40 ET patients and 41 NC were selected for the study of action tremor, and 57 PD patients (29 PD‐RT, 28 PD‐WT) were selected for rest tremor.

### 2.2 MRI sequences

A 3.0T MRI scanner (GE Health Systems, Discovery 750) with an eight‐channel head coil was used to scan all of the participants. Earplugs and foam pads were utilized to decrease noise and head movements for each participant. Fast spoiled gradient recalled sequence was used to capture structural T1 images: repetition time (TR) = 8.1 ms; echo time (TE) = 3.0 ms; inversion time = 450 ms; flip angle = 11°; field of view (FOV) = 256 × 256 mm^2^; voxel size = 1 × 1 × 1 mm^3^; a total of 196 layers. Enhanced T2 star‐weighted angiography sequence was used for reconstructing QSM, and the parameters were as follows: FOV = 240 × 240 mm^2^, TR = 33.7 ms, TE1/space/TE8 = 4.556 ms/3.648 ms/30.092 ms, matrix = 416 × 384, layer thickness = 2 mm, a total of 68 layers. DTI was performed to conduct tractography and scanned by a spin echo‐echo planar imaging sequence: 30 gradient directions; *B* value =0, 1000 s/m^2^; TR = 8000 ms; TE = 80 ms; flip angle = 90°; FOV = 256 × 256; matrix = 128 × 128; slice thickness = 2 mm; a total of 67 layers. At the same time, the opposite phase DTI data were collected to correct the susceptibility distortion.

### 2.3 QSM data processing and segmentation of nuclei

The Susceptibility Tensor Imaging (STI) Suite V3.0 software (Duke University) was used to process phase images on a computer cluster,^21^ as follows: (1) the phase images were unwrapped using the Laplacian approach, which relies solely on the sine and cosine functions of the phase angle;^22^, ^23^ (2) the background phase was eliminated using the V‐SHARP method with the radius of the spherical kernel rising from 0.6 mm at the periphery of the brain to 25 mm toward the center of the brain;^22^ and (3) QSM images were calculated using the streaking artifact reduction for QSM (STAR‐QSM) method.^24^ The mean signal of each individual brain was used as the susceptibility reference.

**FIGURE 1:**
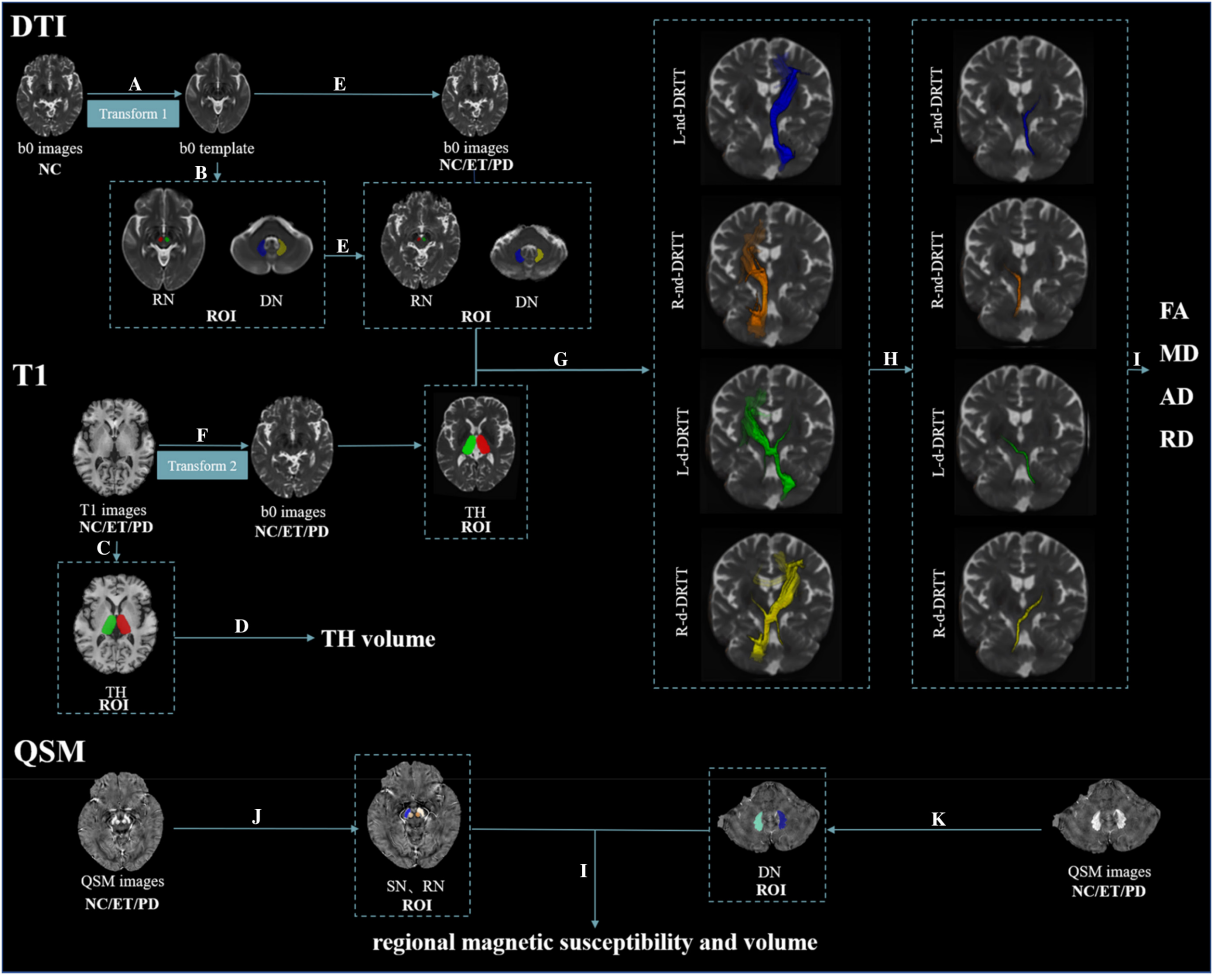
Workflow of the whole processing procedure. (1) Using the “ANTs Multivariate Template Construction” pipeline to create a group‐specific template based on b0 images of NC (A), and sketch DN and RN manually drawn on b0 template image (B). (2) Segmenting the bilateral TH by using FIRST in original T1‐weighted images (C). Meanwhile, the volume parameters of TH are obtained (D). (3) The obtained ROIs (including DN, RN, and TH) were transformed into native b0 images using the invert‐transforms from native b0 template (E) or T1 images (F) to b0 images. (4) The DN was used as seed regions, RN and TH as sequential forced‐order waypoints, and the L‐nd‐DRTT, R‐nd‐DRTT, L‐d‐DRTT, and R‐d‐DRTT were obtained by probabilistic fiber tractography (G). (5) Thresholding (20%) to ensure that only the core of the tracts was chosen (H), and then, the tracts maps were binarized to gain the mean DTI metrics (I). (6) Generated ROIs in native susceptibility maps: automatically divided and manually revised the SN and RN results by using Python‐based (www.python.org) code (J), and manually draw the bilateral DN by using the ITK‐SNAP (www.itksnap.org) (K). Then, the magnetic susceptibility and volume of ROIs are obtained (L). Transform 1: The registration transformation from b0 template to native b0 images using linear and nonlinear registration. Transform 2: the transformation of the registration from native T1 images to native b0 images using linear registration. AD, axial diffusivity; ANTs, Advanced Normalization Tools; DN, dentate nucleus; ET, essential tremor; FA, fractional anisotropy; FIRST, the FMRIB Software Library's Integrated Registration and Segmentation Tool; L‐d‐DRTT, the left decussating dentato‐rubro‐thalamic tract; L‐nd‐DRTT, the left non‐decussating dentato‐rubro‐thalamic tract; MD, mean diffusivity; NC, normal controls; PD, Parkinson's disease; RD, radial diffusivity; R‐d‐DRTT, the right decussating dentato‐rubro‐thalamic tract; RN, red nucleus; R‐nd‐DRTT, the right non‐decussating dentato‐rubro‐thalamic tract; ROI, region of interest; SN, substantia nigra; TH, thalamus.

We used a deep learning‐based end‐to‐end tool (DeepQSMSeg),^25^, ^26^ which was developed by setting the ground truth as the previous manually and semiautomatically segmented data,^27^, ^28^ to segment substantia nigra (SN) and RN (Figure 1J). All the automatically segmented data were checked by an experienced neuroradiologist. And DN was manually segmented using the ITK‐SNAP (www.itksnap.org) by a radiologist who was blinded to the all clinical data (Figure 1K). Then, the regional magnetic susceptibility and volume of the bilateral SN, RN, and DN were extracted (Figure 1L).

### 2.4 DTI and probabilistic fiber tractography

The FMRIB Software Library (FSL) v6.0 package (https://fsl.fmrib.ox.ac.uk/fsl/fslwiki) was used to process DTI images. The following steps were included in the preprocessing procedures: (1) brain extraction with the “BET” tool; (2) the B0‐inhomogeneity distortion correction by using two opposite phase‐encoded images and the “topup” tool; (3) the eddy‐current‐induced handling motion and distortion correction by the “eddy” tool; and (4) fitting of DTI metrics [including fractional anisotropy (FA), mean diffusivity (MD), axial diffusivity (AD), and radial diffusivity (RD)] with “DTIFIT” tool.

The bilateral TH were segmented using FSL's Integrated Registration and Segmentation Tool (FIRST) in native T1‐weighted images (Figure 1C).^29^ Then, the volume of the bilateral TH was extracted (Figure 1D). Meanwhile, the TH structure in the native T1 space was transformed into native DTI space, to perform tractography. A group‐specific template was created by using the Advanced Normalization Tools (ANTs) “antsMultivariateTemplateConstruction” pipeline based on b0 images of NC (Figure 1A). Then, we manually segmented bilateral DN and RN in the template (Figure 1B). The bilateral DN and RN were transformed into native DTI space (Figure 1E) and could be employed in tractography after proper adjustment of the resulting labels in the native DTI space.

The DRTT was calculated using probabilistic fiber tractography and FSL's diffusion toolkit “Fdt”,^30^ which allowed for numerous fibers per voxel and robust detection of crossing fibers. First, diffusion parameters at each voxel were modeled using “bedpostx”, which used Bayesian estimation to build up the distribution of diffusion directions at each voxel. The “probtrackx2” tool was then used to do fiber tracking based on the estimated fiber orientations (with 5000 streamline samples, a step length of 0.5 mm, a curvature threshold of 0.2, and a loop check to exclude tracks that double back on themselves). As previously recommended, four main tracking methods have been referred to: (1) DN‐SCP‐RN;^8^ (2) DN‐RN‐TH;^31^ (3) DN‐RN;^32^ and (4) RN‐cerebral cortex.^33^ Here, we used one of the most commonly applied methods (DN‐RN‐TH) to define DRTTs, where the DN served as the seed regions, with RN and TH as sequential forced‐order waypoints.^8^, ^31^ We defined four fiber bundles on each subject: left d‐DRTT (L‐d‐DRTT) (i.e. originating in the left DN, going through the right RN, and terminating in the right TH), right d‐DRTT (R‐d‐DRTT), left nd‐DRTT (L‐nd‐DRTT) (i.e., originating in the left DN, goes through the left RN, and terminating in the left TH), right nd‐DRTT (R‐nd‐DRTT) (Figure 1G). Then, to ensure that only the core of the tracts was chosen, a threshold of 20% of the maximal value was employed (Figure 1H).^34^ Finally, the tract maps were binarized to obtain the mean DTI metrics (Figure 1I).

To visualize the spatial locations of two types of fiber bundles, all tracts of interest were normalized into the b0 template space using linear interpolation after native thresholding, and the group specificity map of each group was obtained by adding the fiber bundle probability values of all subjects in the same group. Then, we used the MNI space of the ICBM‐152 brain template to observe the spatial relationship between two types of DRTT tracts and the thalamic ventral intermediate nucleus (VIM)^35^ (Figure 3 and Figure S3), and the coordinates of the probability peak points of the fiber bundle in each group of people were obtained (Table 2).

### 2.5 Statistical analysis

All the statistical analyses were conducted by IBM SPSS Statistics v.25.0. Normal distribution of data was checked using the Kolmogorov–Smirnov test. To ensure normally distributed data, DTI metrics (FA, MD, AD, and RD) were logarithmic transformed before statistical analysis.

The fundamental demographic differences in the three groups (ET, PD, and NC) were compared, including Pearson chi‐squared analysis for sex and analysis of variance for age, education, MMSE scores, and total intracranial volume (TIV). Independent samples *t*‐tests were used to assess differences in the UPDRS‐III scores, rest tremor scores, action tremor scores, AR scores, PIGD scores, and disease duration between PD subgroups and between PD and ET. In addition, an independent sample *t*‐test was used to compare the difference in Hoehn–Yahr stage between two PD subgroups.

For image analysis, because a direct comparison of ET with PD involves many confounders (such as AR, PIGD), the following design would help to better analyze the changes and effects of DRT pathway under different tremors. We used general linear models (GLM) to compare the differences in image parameters of DRT pathway between different groups: (1) NC vs ET, with sex and age as covariates, (2) PD‐RT vs PD‐WT, with sex and age as covariates, and (3) NC vs PD, with sex, age, education, and MMSE scores as covariates. In addition, TIV was added as a covariate when comparing nucleus volume between groups. Finally, partial correlation analysis was used to investigate the relationships among clinical behaviors, DTI, and QSM parameters showing significant intergroup differences. Two‐tailed *p* < 0.05 was regarded as statistically significant. Moreover, Bonferroni correction was applied in multiple comparisons to reduce type I error. Thus, *p* < 0.0083 (0.05/6) was judged as statistically significant for regional magnetic susceptibility, *p* < 0.00625 (0.05/8) for the nucleus volume and *p* < 0.0125 (0.05/4) for DTI metrics.

## 3 RESULTS

### 3.1 Demographic and clinical information

We selected 40 ET patients, 57 PD patients (29 PD‐RT, 28 PD‐WT), and 41 NC from the cohort collected by our team from August 2014 to November 2020. As shown in Table 1, no significant difference in age (NC vs ET: *p* = 0.22, NC vs PD: *p* = 0.37, ET vs PD: *p* = 0.65, PD‐RT vs PD‐WT: *p* = 0.96) and TIV (NC vs ET: *p* = 0.59, NC vs PD: *p* = 0.14, ET vs PD: *p* = 0.42, PD‐RT vs PD‐WT: *p* = 0.91) was found among NC, ET, and PD patients. The NC and ET groups had more females (NC vs PD: *p* = 0.04, ET vs PD: *p* < 0.01), higher educational (NC vs PD: *p* < 0.01, ET vs PD: *p* < 0.01), and MMSE scores (NC vs PD: *p* < 0.01, ET vs PD: *p* < 0.01) than the PD group. Considering that these intergroup differences in demographics and clinical symptoms may influence the results, age, and gender were included as covariates in the subsequent analyses of image parameters. In addition, education level and MMSE score were used as covariates in the comparison between the NC and PD groups.

**TABLE 1:** Group demographics and clinical status.

	NC	ET	PD		*p‐*Values			
			PD‐WT	PD‐RT	NC vs ET	NC vs PD	ET vs PD	PD‐WT vs PD‐RT
Number (*n*)	41	40	28	29	–	–	–	–
Sex (male/female)	17/24	12/28	18/10	18/11	0.36	**0**.**04**	**<0**.**01**	1.00
Age (years)	60.81 ± 6.14	58.76 ± 8.31	59.40 ± 7.12	59.46 ± 8.08	0.22	0.37	0.65	0.96
Education (years)	10.59 ± 4.17	8.95 ± 3.51	8.11 ± 4.81	7.79 ± 3.93	0.06	**<0**.**01**	**<0**.**01**	0.79
MMSE scores	28.46 ± 1.73	28.18 ± 2.16	27.18 ± 2.04	26.79 ± 2.76	0.55	**<0**.**01**	**<0**.**01**	0.56
TIV (cm^3^)	1470.39 ± 116.10	1438.89 ± 129.85	1487.84 ± 142.00	1523.60 ± 131.33	0.59	0.14	0.42	0.91
Disease duration (years)	–	12.53 ± 8.13	3.27 ± 2.19	4.94 ± 3.62	–	–	**<0**.**01**	0.27
Hoehn–Yahr stage	–	–	1.98 ± 0.55	1.98 ± 0.57	–	–	–	1.00
UPDRS‐III scores	–	4.68 ± 2.45	17.18 ± 8.91	23.21 ± 11.35	–	–	–	**0**.**03**
Rest tremor scores	–	0.00 ± 0.00	0.00 ± 0.00	2.79 ± 0.86	–	–	–	–
Action tremor scores	–	2.25 ± 0.90	1.21 ± 0.96	1.38 ± 1.05	–	–	–	0.54
AR scores	–	1.30 ± 1.51	12.61 ± 7.16	13.72 ± 8.58	–	–	–	0.60
PIGD scores	–	0.05 ± 0.22	2.25 ± 1.67	2.76 ± 2.03	–	–	–	0.31
TETRAS‐2 scores	–	14.26 ± 8.23	–	–	–	–	–	–
TETRAS upper limbs scores	–	5.66 ± 3.35	–	–	–	–	–	–

*Note*: *p* < 0.05 is considered statistically significant and indicated in bold. Values are expressed as mean ± standard deviation.

Abbreviations: AR, akinetic/rigid; ET, essential tremor; MMSE, the Mini‐Mental State Examination; NC, normal controls; PD‐RT, Parkinson's disease with rest tremor; PD‐WT, Parkinson's disease without rest tremor; PIGD, postural instability/gait disorder; TETRAS, the Essential Tremor Rating Scale; UPDRS, the Unified Parkinson's Disease Rating Scale.

The ET patients had longer disease duration (*p* < 0.01) and higher action tremor (*p* < 0.01) than PD group. Between PD‐RT and PD‐WT patients, there was a statistical difference in UPDRS‐III scores (*p* = 0.03), while there was no significant difference in the action tremor scores (*p* = 0.54), AR scores (*p* = 0.60), PIGD scores (*p* = 0.31), Hoehn–Yahr stage (*p* = 1.00), and disease duration (*p* = 0.27).

### 3.2 Intergroup comparison of iron accumulation in the SN, RN, and DN

**FIGURE 2:**
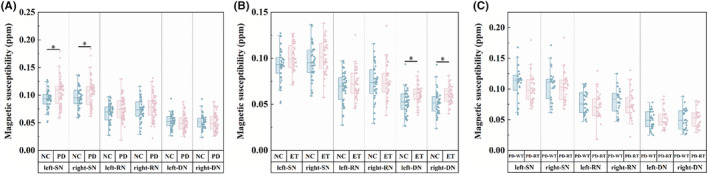
Intergroup differences in magnetic susceptibility in the SN and the nuclei of the DRT pathway using the GLM analysis: adjusted for age, sex, education, and MMSE scores in NC vs PD (A); adjusted for age and sex in PD‐RT vs PD‐WT (B), NC vs ET (C). Bonferroni correction was used for the intergroup comparisons and multiple comparisons. *Statistically significant differences (*p* < 0.0083). DRT pathway, dentato‐rubro‐thalamic pathway; GLM, general linear models; MMSE, the Mini‐Mental State Examination; DN, dentate nucleus; ET, essential tremor; NC, normal controls; PD‐RT, Parkinson's disease with rest tremor; PD‐WT, Parkinson's disease without rest tremor; RN, red nucleus; SN, substantia nigra.

The ET group's magnetic susceptibility was significantly higher in the bilateral DN when compared to the NC group (*p*_left‐DN_ = 0.002, *p*_right‐DN_ = 0.001). The bilateral SN magnetic susceptibility in the PD group was significantly higher than that in the NC group (*p*_left‐SN_ = 0.006, *p*_right‐SN_ = 0.008). No other intergroup difference in regional magnetic susceptibility was observed among groups (Figure 2).

### 3.3 Intergroup comparison in volume of TH, RN, DN, and SN

The bilateral SN volume in the PD group was significantly lower than that in the NC group (*p*_left‐SN_ = 0.006, *p*_right‐SN_ < 0.001). No other intergroup difference in nucleus volume was observed among groups (Figure S2).

### 3.4 Spatial relationship between the d‐DRTT and nd‐DRTT within TH

**FIGURE 3:**
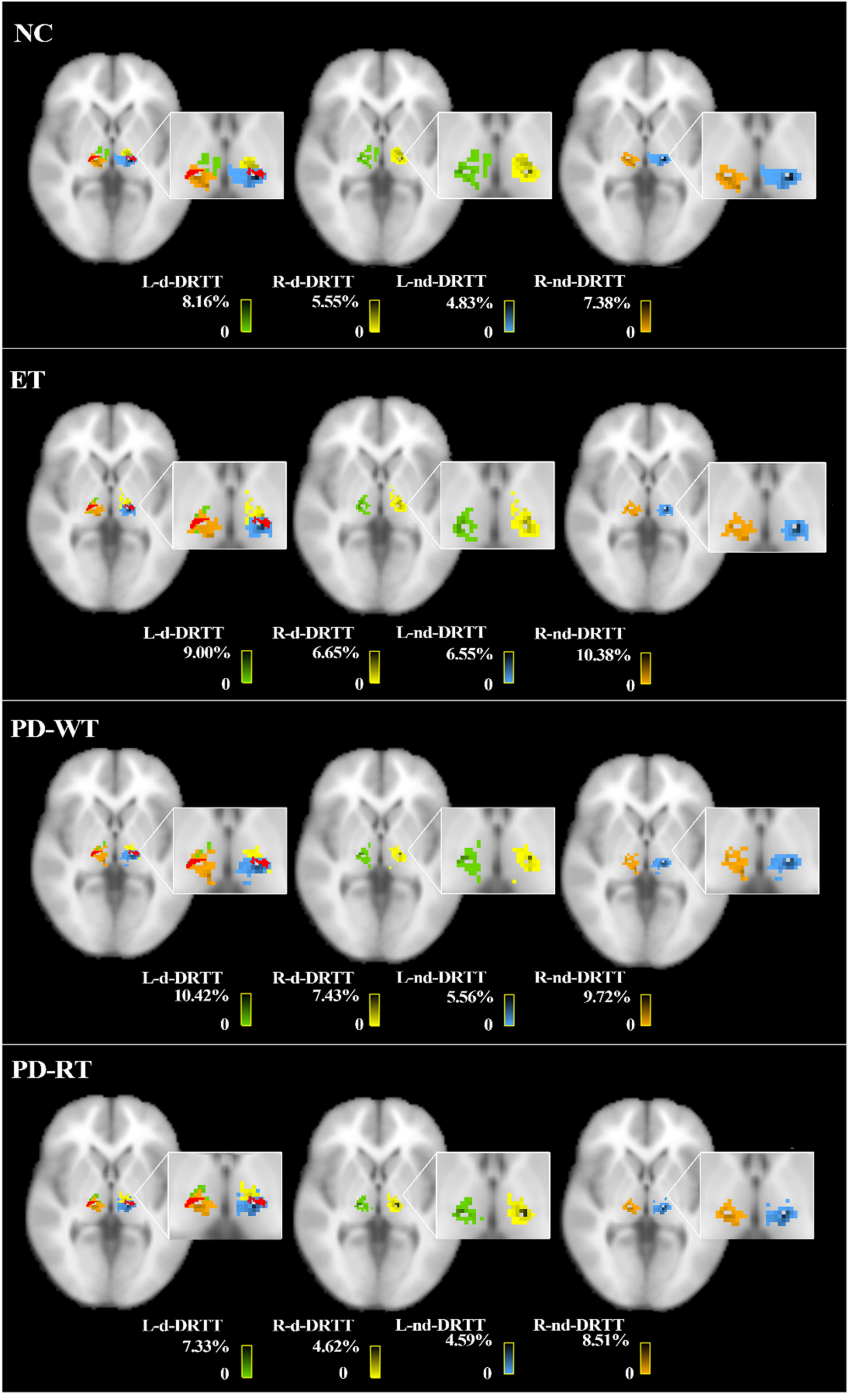
Spatial relationship between the d‐DRTT, nd‐DRTT, and VIM. Axial slices show the L‐nd‐DRTT (blue), R‐nd‐DRTT (orange), L‐d‐DRTT (green), R‐d‐DRTT (yellow), and VIM (red) in the NC, ET, PD‐RT, and PD‐WT groups. After native thresholding, all tracts of interest were normalized into the b0 template space using linear interpolation to produce the population probability maps, and then, we used the MNI space of the ICBM‐152 brain template to observe the relationship between DRTT drop point and VIM. Among them, the color bars reflect the percentage value of the probability of the fiber bundle passing through each voxel divided by the sum of the probability of the fiber bundle passing through all the voxels within the TH, and the darker the color, the larger the percentage value. ET, essential tremor; NC, normal controls; L‐d‐DRTT, the left decussating dentato‐rubro‐thalamic tract; L‐nd‐DRTT, the left non‐decussating dentato‐rubro‐thalamic tract; PD‐RT, Parkinson's disease with rest tremor; PD‐WT, Parkinson's disease without rest tremor; R‐d‐DRTT, the right decussating dentato‐rubro‐thalamic tract; R‐nd‐DRTT, the right non‐decussating dentato‐rubro‐thalamic tract; VIM, the thalamic ventral intermediate nucleus.

**FIGURE 4:**
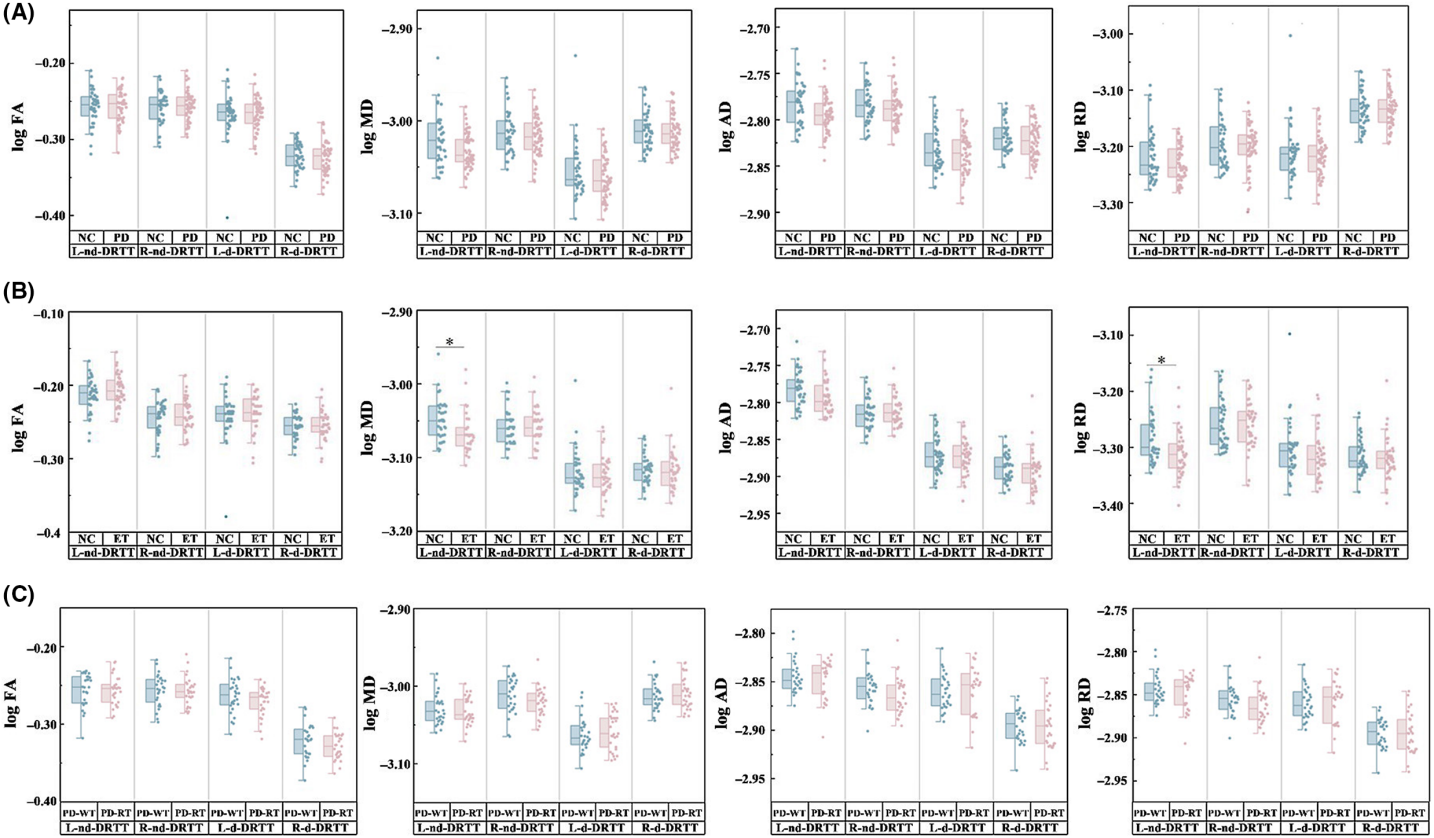
Intergroup differences in diffusion metrics (log FA, log MD, log AD, and log RD) of DRTT using the GLM analysis: adjusted for age, sex, education, and MMSE scores in NC vs PD (A); adjusted for age and sex in NC vs ET (B), PD‐RT vs PD‐WT (C). Bonferroni correction was used for the intergroup comparisons and multiple comparisons. *Statistically significant differences (*p* < 0.0125). AD, axial diffusivity; ET, essential tremor; FA, fractional anisotropy; GLM, general linear models; L‐d‐DRTT, the left decussating dentato‐rubro‐thalamic tract; L‐nd‐DRTT, left nondecussating dentato‐rubro‐thalamic tract; MD, mean diffusivity; MMSE, the Mini‐Mental State Examination; NC, normal controls; PD‐RT, Parkinson's disease with rest tremor; PD‐WT, Parkinson's disease without rest tremor; RD, radial diffusivity; R‐d‐DRTT, the right decussating dentato‐rubro‐thalamic tract; R‐nd‐DRTT, right non‐decussating dentato‐rubro‐thalamic tract.

The tractography data (Figure 4) showed the drop point of the two fiber bundles of interest within the middle layer of VIM (the 36th layer) of each group in the MNI T1 space. In each group, nd‐DRTT and d‐DRTT overlap greatly in TH, and they mainly fall on VIM. Furthermore, the nd‐DRTT is more posterior and medial to d‐DRTT (Figure 3 and Table 2). This spatial position relationship persists at different levels (Figure S3).

**TABLE 2:** The peak probability points for DRTT in the TH (in MNI 2‐mm space).

Group	Peak probability points of DRTT in TH			Total volume (mm^3^)	Overlap volume (mm^3^)
	X (mm)	Y (mm)	Z (mm)		
NC					
L‐nd‐DRTT	‐8	−22	−4	1232	720
R‐d‐DRTT	−8	−18	−4	1224	
R‐nd‐DRTT	10	−24	−4	1288	872
L‐d‐DRTT	10	−20	−4	1248	
ET					
L‐nd‐DRTT	−10	−22	−4	1080	784
R‐d‐DRTT	−12	−16	−2	1280	
R‐nd‐DRTT	10	−22	−4	1232	640
L‐d‐DRTT	14	−18	−2	992	
PD‐WT					
L‐nd‐DRTT	−8	−22	−4	1120	680
R‐d‐DRTT	−12	−18	−2	1008	
R‐nd‐DRTT	10	−22	−4	1000	608
L‐d‐DRTT	12	−20	−4	856	
PD‐RT					
L‐nd‐DRTT	−8	−22	−4	1256	832
R‐d‐DRTT	−14	−18	−2	1224	
R‐nd‐DRTT	10	−22	−4	1200	696
L‐d‐DRTT	12	−18	−4	1064	

*Note*: Total volume: The extent of the fiber bundle landing on the thalamus. Overlap volume: overlap range of nd‐DRTT and d‐DRTT drop points on the same side of the thalamus.

Abbreviations: ET, essential tremor; L‐d‐DRTT, the left decussating dentato‐rubro‐thalamic tract; L‐nd‐DRTT, the left non‐decussating dentato‐rubro‐thalamic tract; NC, normal controls; PD‐RT, Parkinson's disease with rest tremor; PD‐WT, Parkinson's disease without rest tremor; R‐d‐DRTT, the right decussating dentato‐rubro‐thalamic tract; R‐nd‐DRTT, the right non‐decussating dentato‐rubro‐thalamic tract; TH, thalamus.

### 3.5 Intergroup comparison in diffusion metrics of DRTT

Compared with NC, ET patients had significantly lower log MD and log RD in the left nd‐DRTT (L‐nd‐DRTT) (*p*_L‐nd‐DRTT log MD_ = 0.004, *p*_L‐nd‐DRTT log RD_ = 0.003), while no difference in any other diffusion metrics. No difference in any other diffusion metrics was found between PD and NC or among PD subgroups (Figure 4).

### 3.6 Relationship of DTI, QSM parameters, and clinical symptoms

In the ET group, increased TETRAS scores in upper limbs (*r* = −0.446, *p* = 0.004) and TETRAS scores in right upper limbs (*r* = −0.411, *p* = 0.008) were significantly associated with decreasing the log MD in L‐nd‐DRTT, while TETRAS scores in right upper limbs (*r* = −0.318, *p* = 0.045) were significantly negative relationships with the log RD (Figure S4). But there was no significant association between tremor scores and magnetic susceptibility in the DN. There was no significant relationship between tremor ratings and magnetic susceptibility in the PD group. Furthermore, in ET, the log RD of L‐nd‐DRTT was correlated with the magnetic susceptibility of DN (*r* = 0.322, *p* = 0.042; Figure S4).

## 4 DISCUSSION

The classical tremor pathway of two types of the tremor was examined thoroughly by taking the advantages of multi‐modality MRI. Three main findings were indicated here: (1) in ET, the iron content in DN was significantly higher than in NC; (2) the nd‐DRTT and d‐DRTT overlap a lot in the TH, mainly on VIM, but nd‐DRTT is more posterior and medial to d‐DRTT; and (3) the MD and RD of the L‐nd‐DRTT in ET were significantly lower than that in NC, and had a negative relationship with tremor severity.

As a primary beginning point of the DRT pathway, bilateral DN in ET patients had significantly increased iron concentration when compared to NC. According to a growing body of researches, the overactivation of DN is closely related to the synthesis of excitatory neurotransmitters such as glycine and glutamate,^36^ where the synthesis of neurotransmitters requires the sufficient participation of iron.^37^, ^38^ In ET patients, as mentioned in the common part of the GABA hypothesis and the degenerative hypothesis, the reduction in the number and shape of Purkinje cells leads to weakened inhibition on DN, resulting in overactivation of DN, which is widely accepted as an important cause of abnormal action tremor.^39^, ^40^, ^41^, ^42^, ^43^ Therefore, the increase in iron content in DN may be a manifestation of the synthesis of neurotransmitters by the uptake of more iron during the overactivation of DN, which may be related to the overactivation of abnormal action tremor, further supporting the common part of the GABA hypothesis and the degenerative hypothesis: the overactivation of DN after disinhibition. Consistently, previous studies have reported excessive iron deposition in DN in ET patients.^44^ However, in other studies of iron deposition in the nuclei of ET patients, no excessive iron deposition in DN was found.^45^, ^46^ This may be related to the different imaging techniques used: R2* rates were used for analysis in the previous two papers,^45^, ^46^ whereas QSM has been widely shown to be more sensitive and robust.^9^, ^47^ Furthermore, the inconsistent results may also be due to differences in the clinical characteristics of enrolled patients, such as rest tremor^45^ and long‐term medication.^46^ In addition, the difference in sample size may also be one of the reasons for the difference in results. In summary, in the present study, increased iron content in DN may represent abnormally overactivation of DN, which may be further related to the development of action tremor.

ET patients had significantly lower MD and RD in the L‐nd‐DRTT. In the adult brain, nerve fibers still have plasticity, which may be achieved through changes in axon diameter, axon density, and myelin sheath number.^48^ RD value is closely related to myelination, and is a specific evaluation index of demyelination and myelin regeneration.^49^, ^50^ The decreased RD may represent myelin regeneration and may be a manifestation of fiber bundle activation.^48^, ^51^ And MD is the average value of diffusivity in the three diffusion directions, which may be influenced to some extent affected by the RD value. Thus, in ET patients with action tremor, a decrease in RD and MD of L‐nd‐DRTT may reflect nerve fiber bundle remodeling, especially fiber myelination. This may be a signal of pathological overactivation of the DRT pathway, which is consistent with the GABA hypothesis and the degenerative hypothesis that the overactivation of DN leads to core changes in the DRT pathway overactivation.^2^, ^5^, ^6^, ^52^ In addition, the decrease in MD and RD in L‐nd‐DRTT was correlated with increased action tremor severity, further supporting this hypothesis. Consistent with us, one study found increased connectivity between the cerebellum, TH, and cortex in ET patients,^53^ and another found reduced GABA function and overactivity of the DRT pathway in ET patients.^52^ However, in another study, the researchers found that ET had higher RD in the cerebellar peduncles and degeneration of white matter fibers compared with PD.^7^ The explanation for this contradicting conclusion may be that the region of interest in this study was not a single fiber bundle but contains all fibers passing through the cerebellar peduncles, so the results are mixed with changes in different fiber bundles. In general, there may be fiber remodeling in nd‐DRTT, which may be related to the development of action tremor.

Moreover, the nd‐DRTT and d‐DRTT were found to be overlapped greatly in the TH, particularly on VIM. For medication‐resistant patients, deep brain stimulation and MRI‐guided focused ultrasound targeting the VIM have been shown to be effective in controlling tremor.^54^, ^55^, ^56^ However, the precise location of therapeutic targets is still controversial, but it plays an important role in efficacy, prognosis, and mitigation of toxic and side effects.^57^ In our study, we found an association between nd‐DRTT and action tremor. In addition, we observed that there is a difference in the spatial location between them: The nd‐DRTT is located posteriorly and medially to the d‐DRTT, which was largely supported by the previous literature.^8^, ^58^ Therefore, these findings indicated that targeting the posterior and medial regions of VIM may be more effective in preventing DRTT hyperactivity. Of course, future studies with larger sample sizes in a prospective and multicenter design would further promote clinical translation.

For the downstream of the DRT pathway, there is no significance in the volume of the bilateral TH and RN, the same as the iron content in RN. This result may be related to the mild severity of tremor in the ET patients. Both TH and RN belong to the downstream of DRT pathway, and their damage may occur later.

In summary, we mainly found changes in the two main components of DRT pathway in ET patients: (1) increased iron deposition of bilateral DN; (2) decreased MD and RD of L‐nd‐DRTT. Specifically, iron deposition in DN may be related to the overactivation of DN, while the decrease in MD and RD in nd‐DRTT may be related to fiber remodeling, both of which represent the possibility of overactivation of DRT pathway. In addition, a potentially interesting phenomenon in ET patients was observed, that was the iron content of DN was positively correlated with RD of nd‐DRTT. This is different from the excessive iron deposition in DN and decreased MD and RD in nd‐DRTT found in ET patients, which may mean that the changes in iron and fiber bundles may vary from disease stages that means when iron deposition reaches a certain extent in the future, it will have a negative impact on nerve fiber bundles.^59^ Therefore, future multi‐phase longitudinal studies are necessary to confirm our findings.

However, when comparing PD with NC, significantly increased iron content and decrease volume were only found in bilateral SN, which is consistent with the fact that iron accumulation in the SN characterizes PD.^9^, ^60^ In the DRT pathway, there were no significant variations in nuclei or nerve fibers between PD and NC, or between PD‐RT and PD‐WT. Consistent with us, no significant difference in cerebellar microstructure between ET with and without rest tremor was found previously,^61^ one explanation is that networks other than cerebellar circuits may play a role in ET with resting tremor. Another study on PD tremor found that compared with NC and PD patients without tremor, PD patients with tremor had extensive brain fiber cross‐section widening, which may be due to the compensation of fiber bundles in PD tremor.^62^ Therefore, for rest tremor, the cause may be related to changes in neural circuits outside the DRT pathway, and a wider range of neural circuits are warranted to be conducted in future studies.

There are still some limitations in our study: First, the diagnostic criteria for ET depend on the Movement Disorders Society Statement,^10^ and this statement is currently vague: they do not explicitly state which examination items are required to diagnose ET and they do not specify a minimum cut‐off value for action tremor. Thus, ET‐related examination items need to be verified in the future studies. Second, to match patients with both tremor types, we only counted the upper limb tremor scores, so future extension of this result needs to be done with caution. Third, because of the heterogeneity of motor symptoms in PD and ET, the sample size of the patients with pure rest tremor is very small, making it difficult to address the hypothesis in this way. Alternatively, in order to reduce the influence of the symptoms other than tremor as much as possible, we used PSM to highlight the relatively independent effects of rest tremor (PD‐WT vs PD‐RT) and action tremor (ET vs NC) while other factors were well‐matched. However, future studies remain warranted to validate our findings by using larger sample size and including patients with pure tremors. Furthermore, even though all patients receiving antiparkinsonian pharmaceuticals were stopped for more than 12 hours in this trial, the medicinal effects on brain function could not be entirely avoided, such as the long‐duration response to levodopa in PD patients.^63^

In summary, we systematically explored each component of the DRT pathway and revealed DN iron deposition and nd‐DRTT remodeling in action tremor, which was indicating the hypothesis of pathological overactivation of action tremor. In addition, no changes in the DRT pathway were found in rest tremor, suggesting that changes in the DRT pathway may be specific for action tremor.

## AUTHOR CONTRIBUTIONS

Xiaojie Duanmu contributed to the conception, organization, and implementation of the research project, the design and implementation of the statistical analysis, and writing of the first draft of the manuscript. Jiaqi Wen contributed to the conception, organization, and implementation of the research project, the design of the statistical analysis, and writing of the first draft of the manuscript. Sijia Tan contributed to the conception and organization of the research project, the design and review and comment of the statistical analysis, and the review and comment of the manuscript. Tao Guo and Cheng Zhou contributed to the conception, organization of the research project, the review and comment of the statistical analysis, and the review and comment of the manuscript. Haoting Wu, Jingjing Wu, Zhengye Cao, Xiaocao Liu, Jingwen Chen, Chenqing Wu, Jianmei Qin, and Luyan Gu contributed to the conception, organization of the research project, the implementation of the statistical analysis, and review and comment of the manuscript. Yaping Yan, Baorong Zhang, and Minming Zhang contributed to the review and comment of the research project and review and comment of the manuscript. Xiaojun Guan and Xiaojun Xu contributed to the conception and organization of the research project, design and review and comment of the statistical analysis, and writing of the first draft and review and comment of the manuscript.

## FUNDING INFORMATION

This work was supported by the Natural Science Foundation of Zhejiang Province (Grant nos. LY22H180002 and LQ21H180008), the National Natural Science Foundation of China (Grant nos. 82171888, 82001767, 82271935, 82202091, and 81971577), and the 13th Five‐year Plan for National Key Research and Development Program of China (Grant no. 2016YFC1306600).

## CONFLICT OF INTEREST STATEMENT

The authors declare no conflicts of interest.

## PATIENT CONSENT STATEMENT

All patients signed an informed consent approved by the Medical Ethics Committee of the Second Affiliated Hospital of Zhejiang University School of Medicine.

## Supporting information


Data S1.

## Data Availability

The materials used and/or analyzed during the current study are available from the corresponding author upon reasonable request.
