# Supplementary material for: Aberrant dentato‐rubro‐thalamic pathway in action tremor but not rest tremor: A multi‐modality magnetic resonance imaging study
Source: CNS Neurosci Ther. 2023 Jul 5;29(12):4160–71. doi: 10.1111/cns.14339 (PMC10651946; doi:10.1111/cns.14339)
Supplement: Supplementary file 1 — Data S1. [file CNS-29-4160-s001.docx]

**Supplementary Materials**

**Aberrant dentato-rubro-thalamic pathway in action tremor but not rest tremor: a multi-modality magnetic resonance imaging study**

Xiaojie Duanmu^1#^, Jiaqi Wen^1#^, Sijia Tan^1^, Tao Guo^1^, Cheng Zhou^1^, Haoting Wu^1^, Jingjing Wu^1^, Zhengye Cao^1^, Xiaocao Liu^1^, Jingwen Chen^1^, Chenqing Wu^1^, Jianmei Qin^1^, Luyan Gu^2^, Yaping Yan^2^, Baorong Zhang^2^, Minming Zhang^1^, Xiaojun Guan^1*^, Xiaojun Xu^1*^

^1.^ Department of Radiology, The Second Affiliated Hospital, Zhejiang University School of Medicine, Hangzhou, China

^2.^ Department of Neurology, The Second Affiliated Hospital, Zhejiang University School of Medicine, Hangzhou, China

#: These authors contribute equally to this study.

^*^ Correspondence to: Dr. Xiaojun Xu (E-mail: [xxjmailbox@zju.edu.cn](mailto:xxjmailbox@zju.edu.cn)) and Xiaojun Guan (E-mail: xiaojunguan1102@zju.edu.cn) Department of Radiology, The Second Affiliated Hospital, Zhejiang University School of Medicine, No.88 Jiefang Road, Shangcheng District, Hangzhou, China, 31009, Phone: 86-0571-87315255, Fax: 86-0571-8735255.

#
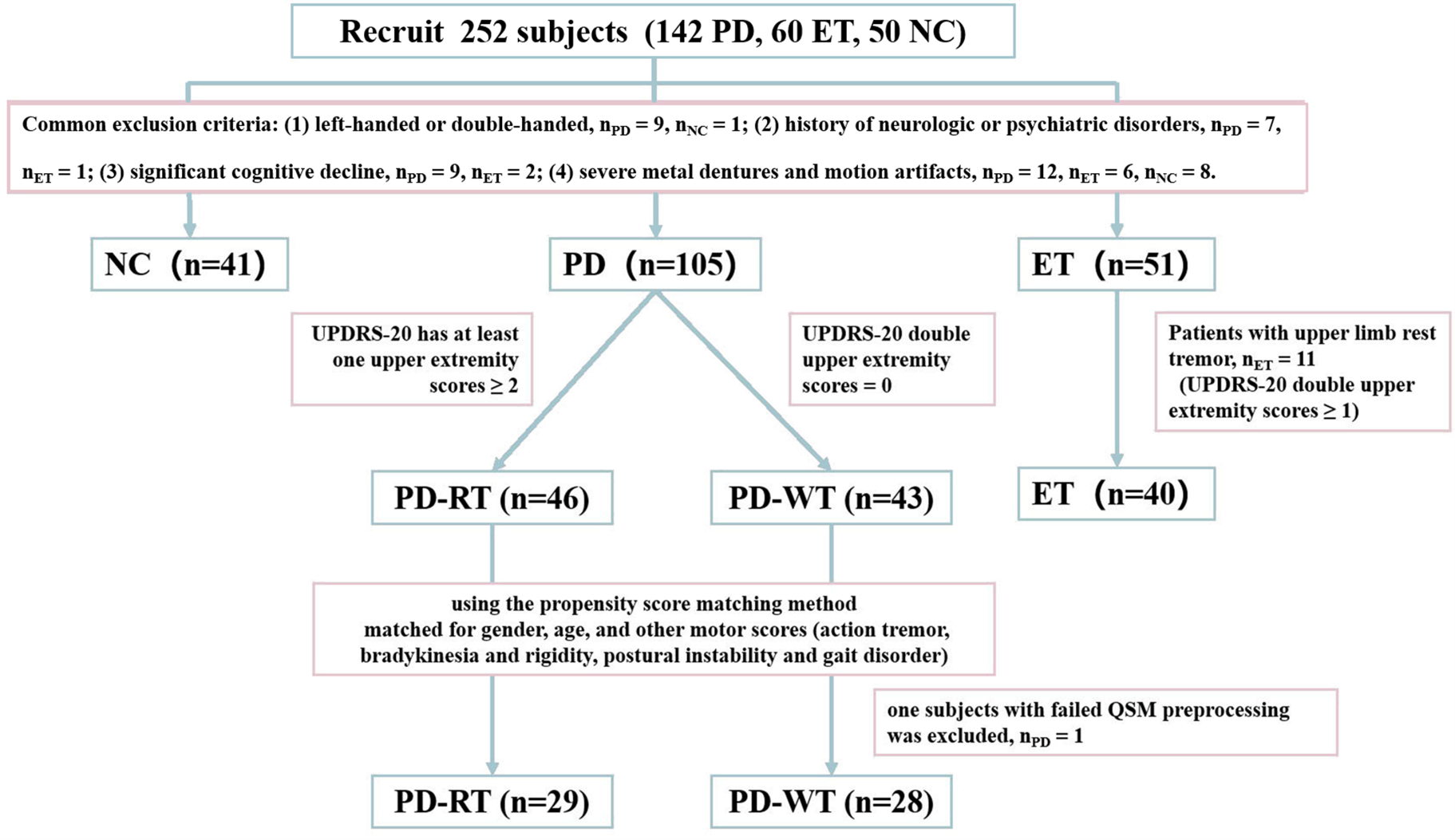
Supplementary Figures

**Supplementary Figure 1.** Inclusion and exclusion processes





**Supplementary Figure 2.** Intergroup differences in the volumes of the SN and the nuclei of the DRT pathway using the GLM analysis: adjusted for age, sex, education, MMSE scores and TIV in NC vs PD (A); adjusted for age, sex, and TIV in PD-RT vs PD-WT (B), NC vs ET (C). Bonferroni correction was used for the intergroup comparisons and multiple comparisons. * indicates statistically significant differences (*P* < 0.00625).

DRT pathway = dentato-rubro-thalamic pathway; GLM = general linear models; MMSE = the Mini-Mental State Examination; TIV = total intracranial volume; TH = thalamus; SN = substantia nigra; RN = red nucleus; DN = dentate nucleus; PD-RT = Parkinson’s disease with rest tremor; PD-WT = Parkinson’s disease without rest tremor; ET = essential tremor; NC = normal controls


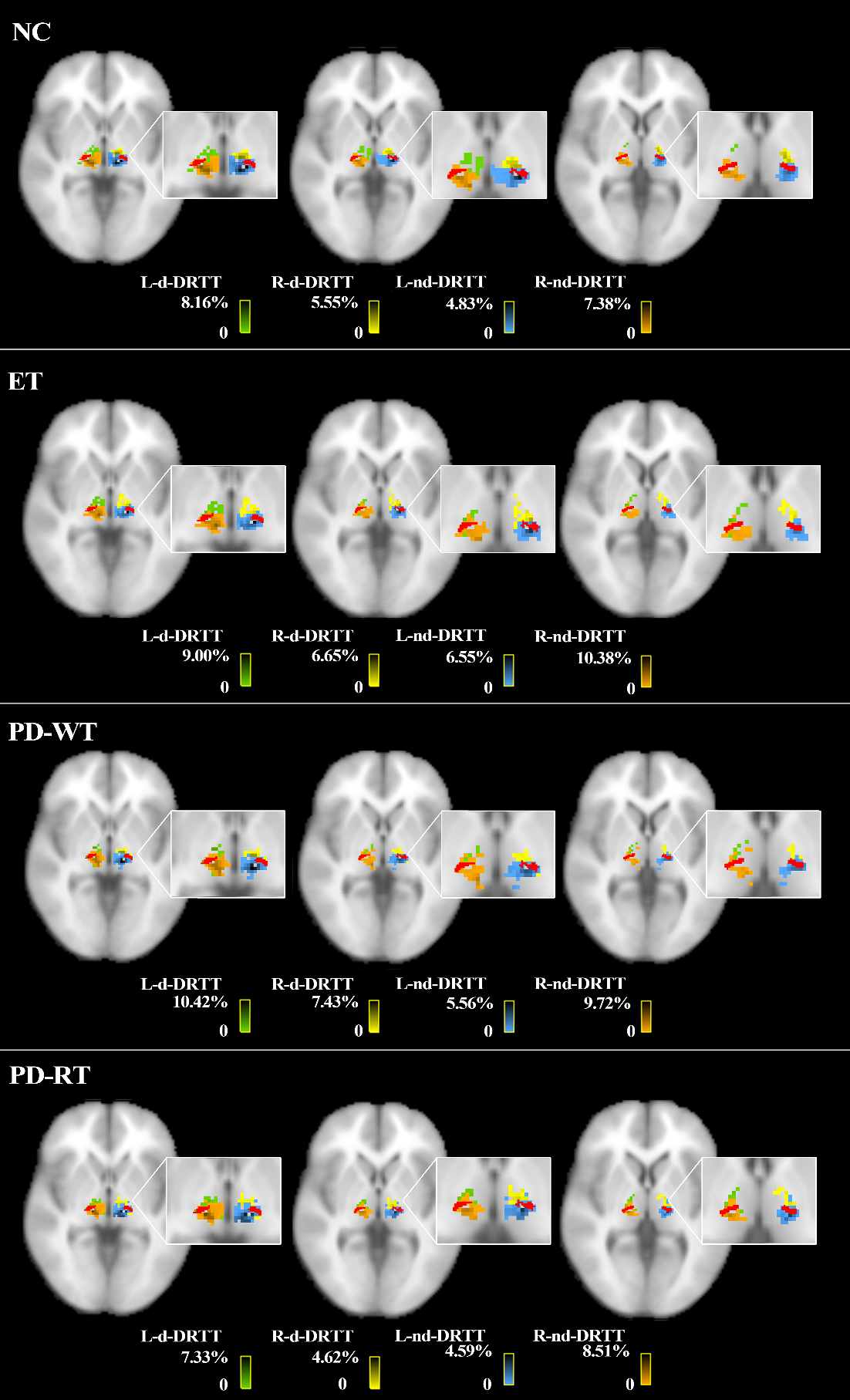
­

­ **Supplementary Figure 3.** Spatial relationship between the d-DRTT, nd-DRTT, and VIM. Axial slices (34-36 layers) show the left nd-DRTT (blue), right nd-DRTT (orange), left d-DRTT (green), right d-DRTT (yellow), and VIM (red) in the NC, ET, PD-RT, and PD-WT groups. After native thresholding, all tracts of interest were normalized into the b0 template space using linear interpolation to produce the population probability maps, and then we used the MNI space of the ICBM-152 brain template to observe the relationship between DRTT drop point and VIM. Among them, the colorbars reflect the percentage value of the probability of the fiber bundle passing through each voxel divided by the sum of the probability of the fiber bundle passing through all the voxels within the TH, and the darker the color, the larger the percentage value.

d-DRTT = decussating dentato-rubro-thalamic tract; nd-DRTT = non-decussating dentato-rubro-thalamic tract; VIM = the thalamic ventral intermediate nucleus; NC = normal controls; ET = essential tremor; PD-RT = Parkinson’s disease with rest tremor; PD-WT = Parkinson’s disease without rest tremor





**Supplementary Figure 4.** Partial correlation analysis (adjusted for age and sex). In ET patients, the negative correlations between log MD in L-nd-DRTT and tremor severity were assessed by the TETRAS upper limbs scores and TETRAS right upper limbs scores (A); the negative correlations between log RD in L-nd-DRTT and tremor severity were assessed by the TETRAS right upper limbs scores (B); the positive correlations between magnetic susceptibility in left-DN and log RD in L-nd-DRTT (C).

MD = mean diffusivity; RD = radial diffusivity; L-nd-DRTT = left non-decussating dentato-rubro-thalamic tract; DN= dentate nucleus; TETRAS = the Essential Tremor Rating Scale; ET = essential tremor
